# Impact of Pickling Pretreatment on the Meat Quality of Frozen–Thawed Freshwater Drum (*Aplodinotus grunniens*)

**DOI:** 10.3390/foods14223845

**Published:** 2025-11-10

**Authors:** Wanwen Chen, Sharifa Mohamed Miraji, Lanxian Yang, Jian Wu, Xueyan Ma, Wu Jin, Liufu Wang, Yufeng Wang, Pao Xu, Hao Cheng, Haibo Wen

**Affiliations:** 1 Wuxi Fisheries College, Nanjing Agricultural University, Wuxi 214081, China; chenwanwen@ffrc.cn (W.C.); sherymiraj@gmail.com (S.M.M.); wangliufu@ffrc.cn (L.W.);; 2 Key Laboratory of Integrated Rice-Fish Farming Ecology, Ministry of Agriculture and Rural Affairs, Freshwater Fisheries Research Center, Chinese Academy of Fishery Sciences, Wuxi 214081, China; 17673919073@163.com; 3 Sino-US Cooperative International Laboratory for Germplasm Conservation and Utilization of Freshwater Mollusks, Freshwater Fisheries Research Center, Chinese Academy of Fishery Sciences, Wuxi 214081, China; 4 Department of Fisheries Development and Marine Resources, Ministry of Blue Economy and Fisheries, P.O. Box 774, Zanzibar 70471, Tanzania; 5 State Key Laboratory of Food Science and Resources, Jiangnan University, Wuxi 214122, China; 6 School of Food Science and Technology, Jiangnan University, Wuxi 214122, China

**Keywords:** freshwater drum, pickling treatment, frozen, texture, water-holding capacity, myofibrillar protein

## Abstract

The freshwater drum (*Aplodinotus grunniens*) is a promising aquaculture species due to its strong environmental adaptability, tolerance to low temperatures, rapid growth rate, high nutritional value, high-quality texture (garlic-clove-shaped flesh), and absence of intermuscular bones. Nevertheless, processing technologies related to freshwater drum remain largely unexplored. Salting pretreatment serves as a viable strategy for enhancing the quality attributes of frozen fish products. This study investigated the effects of different sodium chloride (NaCl) pickling concentrations (0.25, 1, and 3 mol/L) on the physicochemical properties and quality attributes of frozen–thawed freshwater drum (*Aplodinotus grunniens*). Results indicated that elevated NaCl concentrations (1–3 mol/L) significantly (*p* < 0.05) shortened the transit time through the maximum ice crystal formation zone during freezing, effectively mitigating structural damage to myofibrillar networks. As the NaCl concentration increased from 0 to 3 mol/L, the water content decreased from 71.26 ± 0.22% to 68.64 ± 0.50%, while the salt content increased from 0.31 ± 0.01% to 8.46 ± 0.12%. Pickling pretreatment markedly enhanced water-holding capacity and improved texture profiles, including hardness, springiness, gumminess, and chewiness. Histological analysis revealed preserved myofibril integrity in high-salt-treated samples, supported by reduced fluorescence intensity of myofibrillar proteins, indicating mitigated freeze-induced denaturation. Low-field NMR confirmed salt-induced redistribution of water states, with decreased free water proportion. Our results identify that pretreatment with NaCl at concentrations ≥ 1 mol/L is an effective strategy to preserve the post-thaw quality. Due to 3 mol/L NaCl resulting in a relatively high salt content, 1 mol/L NaCl pretreatment is more suitable for maintaining the quality of freeze–thawed freshwater drums.

## 1. Introduction

The freshwater drum (*Aplodinotus grunniens*) is one of the few *sciaenid* species that exclusively inhabit freshwater environments throughout its life cycle [1]. As a widely distributed aquaculture species across North and Central America, our research group has successfully introduced this species, motivated by its superior commercial qualities, including thick dorsal musculature devoid of intermuscular bones, exceptional meat texture, and high nutritional value. This initiative has achieved a major advancement in artificial propagation and rearing techniques since 2019 [2]. The evolution of meat quality parameters during processing and storage exhibits significant species-specific variation [3]. Nevertheless, studies focusing on processing and preservation of the freshwater drum remain remarkably scarce. Similar to most freshwater species, freshwater drum exhibits high susceptibility to spoilage during storage, primarily attributed to its high moisture content and abundant protein matrix. These intrinsic properties accelerate chemical deterioration (e.g., lipid oxidation, protein denaturation) and promote microbial proliferation, leading to irreversible quality loss and potential food safety concerns [4].

Freezing is widely recognized as one of the most effective preservation techniques for maintaining the quality of fresh muscle foods by significantly retarding deterioration processes [5]. During the freezing process, water crystallization in muscle tissue initiates when temperatures drop below −1 °C. As the temperature continues to decrease, the proportion of frozen water increases significantly, with over 90% of muscle water transitioning to the frozen state by −20 °C [6]. However, temperature fluctuations during freezing would cause recrystallization of ice, which mechanically damages cellular structures and muscle fibers, leading to compromised product quality, manifesting as reduced water-holding capacity, altered texture, surface discoloration, and flavor degradation [7]. The freezing-induced denaturation mechanism of aquatic products involves three interrelated pathways: (1) ice crystal nucleation and growth, (2) protein structural destabilization, and (3) lipid oxidative degradation, collectively contributing to quality deterioration during frozen storage [8]. Previous studies have demonstrated that both small-molecule cryoprotectants (e.g., salts, polyols, sugars) and macromolecular cryoprotectants (e.g., antifreeze proteins, polysaccharides, proteins) can effectively stabilize the physicochemical properties and microstructural integrity of frozen aquatic products, thereby preserving meat quality and nutritional value [9]. Nevertheless, the efficacy of cryoprotectants in modulating ice crystal formation and preserving the quality of thawed fish exhibits species-specific variation [10].

Sodium chloride (NaCl) serves as both a traditional preservative and a multifunctional food additive in meat processing, effectively enhancing organoleptic properties while extending shelf life [11]. It is well-established that NaCl induces concentration-dependent structural modifications in myofibrillar proteins, which subsequently regulate three critical quality parameters of muscle foods, including water-holding capacity, textural properties, and color characteristics of muscle foods [12]. Jiang et al. reported that brining tuna meat for 5 h improved water-holding capacity by 20–25% and shear force resistance by 15%, while 1.0 M NaCl treatment reduced freeze-induced myofibrillar fragmentation, preserving muscle fiber integrity post-thawing [13]. These effects exhibit significant variation based on salting parameters, with salt concentration being the predominant determinant [14]. An optimal NaCl concentration facilitates myofibrillar protein solubilization [15]. It was revealed that significant alterations in protein secondary structure and surface hydrophobicity were strongly correlated with declines in water-holding capacity and textural deterioration of bighead carp filets during the initial storage period (4–12 weeks) [16]. Salt ions interact with charged residues on proteins, altering surface charge distribution and enhancing solubility and hydrophilicity. Concurrently, ion-dipole interactions between Na^+^/Cl^−^ and water molecules disrupt hydrogen bonding networks, inhibiting ice crystal nucleation and growth during frozen storage [17]. Due to the well-established association between excessive sodium intake and increased risks of hypertension and cardiovascular diseases, lightly salted fish products (≤3% NaCl) have gained significant popularity in the growing consumer market. These products offer convenience, as they require no desalination or rehydration prior to consumption or cooking [4].

While extensive research has examined the freezing of pre-salted meat products, most studies have focused on long-term frozen storage (>4–8 weeks) and its impact on physicochemical and microstructural properties of lightly salted aquatic tissues. In contrast, little is known about the short-term freezing behavior of freshwater drum (*Aplodinotus grunniens*), a recently domesticated aquaculture species with unique muscle structure and high commercial potential. However, this gap limits our understanding of how salt concentration influences muscle structure, water distribution, and textural properties immediately after freezing. Therefore, the present study systematically investigates the impact of different NaCl concentrations (0–3 mol/L) on short-term frozen–thawed quality parameters in freshwater drum (*Aplodinotus grunniens*), including freezing kinetics, water content, salt content, water activity, pH, color, water-holding capacity, water distribution, histological integrity, texture, and protein structural changes. This work provides new insight into the mechanisms by which NaCl concentration regulates freezing kinetics and protein–water interactions in this species. We hypothesized that elevated NaCl concentrations would enhance freezing efficiency and protect muscle integrity. These comprehensive analyses aim to establish fundamental correlations between salt concentration and product quality, thereby providing a scientific basis for optimizing salting pretreatment and improving short-term frozen storage quality in freshwater fish processing. Ultimately, this study provides new understanding of how controlled salting can help preserve the physicochemical and structural integrity of freshwater fish during freezing, offering both scientific and practical value for future processing applications.

## 2. Materials and Methods

### 2.1. Sample Preparation and Salting

Freshwater drums (*Aplodinotus grunniens*), with an average body weight of 1533.9 ± 99.7 g and total length of 45.3 ± 2.9 cm, were provided by the Freshwater Fisheries Research Center of the Chinese Academy of Fishery Sciences. Live fish were collected from ponds and transported to the laboratory under oxygenated conditions. The fish were killed, eviscerated, and washed, with all processing completed within 30 min. The dorsal muscle meat was collected and cut into cubes of 4.0 cm × 2.0 cm × 1.0 cm using a stainless steel scalpel.

The filets were randomly allocated into four experimental groups, including three treatment groups and one control group. For salt treatment, muscle samples were immersed in NaCl solutions at concentrations of 0.25, 1.0, or 3.0 mol/L using a solid-to-liquid ratio of 1:3 (*w*/*w*). Static salting was performed at 4 °C for 2 h. Following the salting process, samples were surface-dried with filter paper before freezing. Then all the samples were packed in polyethylene bags and frozen at −20 °C for 5 days. The untreated (non-marinated) muscle samples served as the control group. Prior to analysis, all frozen samples were thawed at 4 °C for approximately 12 h to ensure complete thawing under controlled conditions.

### 2.2. Determination of Freezing Characteristics

The freezing curves of cured and control (uncured) fish samples were determined using a freezing point determination method [18]. Temperature monitoring was conducted by inserting a calibrated temperature probe (accuracy ± 0.1 °C) to a depth of 1.0 cm in the geometric center of each sample. Samples were then subjected to freezing at −20 °C in a refrigerator. Temperature data were recorded at 1 min intervals throughout the freezing process with a measurement accuracy of ±0.1 °C. The resultant time–temperature profiles were subsequently analyzed to generate characteristic freezing curves for each treatment group.

### 2.3. Quality Characteristics Analysis

#### 2.3.1. Water Content and Salt Content Determination

The water content of freshwater drum muscle samples was determined by oven-drying at 105 °C to constant mass according to the AOAC official method 950.46 [19]. The salt content of freshwater drum muscle samples was analyzed following the standard method GB 5009.44-2016 [20].

#### 2.3.2. Water Activity Determination

The water activity (aw) of freshwater drum muscle samples was determined using a LabMaster-aw neo water activity meter (Novasina AG, Lachen, Switzerland) following the manufacturer’s protocols. Briefly, the freshwater muscle samples were trimmed into uniform pieces and placed in a pre-cleaned sample dish. The dish was then inserted into the water activity meter’s measurement chamber. Measurements were conducted for a minimum of 15 min until equilibrium was reached, and the final aw value was recorded. Prior to measurements, the instrument was calibrated using a saturated sodium chloride solution to ensure measurement accuracy.

#### 2.3.3. pH Measurement

Approximately 2 g of freshwater drum muscle samples were homogenized with 20 mL of distilled water using a DS-130 handheld ultra-homogenizer (WIGGENS, Straubenhardt, Germany) at 10,000 rpm for 3 min. The pH of the resulting homogenate was measured using an AB23PH/F pH meter (OHAUS, Parsippany, NJ, USA). Measurements were recorded once stable readings were obtained (±0.01 pH unit variation over 30 s) and performed in triplicate.

#### 2.3.4. Color Detection

Color parameters were measured using a HunterLab ColorQuest XE spectrophotometer (Hunter Associates Laboratory, Inc., Reston, VA, USA). The instrument was calibrated prior to analysis using a certified white reflectance standard tile. Each sample was measured at three random locations to determine L* (lightness), a* (redness/greenness), and b* (yellowness/blueness) values.

#### 2.3.5. Water-Holding Capacity

The water-holding capacity (WHC) of frozen freshwater drum filets was evaluated through thawing loss, centrifuging loss, and cooking loss measurements, following established methodologies with slight modifications [21].

##### Thawing Loss Determination

Fish muscle samples were weighed before (W_1_) and after (W_2_) thawing, with surface moisture removed using filter paper. The thawing loss was calculated as:

(1) $$\mathrm{Thawing}\;\mathrm{loss}\;(\%)=\frac{W_{1}-W_{2}}{W_{1}}\times 100\;$$

##### Centrifugation Loss Determination

Thawed fish muscle (W_3_) was wrapped in filter paper to remove surface moisture, then centrifuged at 3000× *g* for 10 min at 4 °C. The post-centrifugation weight (W_4_) was recorded, and the loss was calculated as:

(2) $$\mathrm{Centrifugation}\;\mathrm{loss}\;(\%)=\frac{W_{3}-W_{4}}{W_{3}}\times 100\;$$

##### Cooking Loss Determination

The filter paper was used to absorb surface moisture from the thawed fish muscle to obtain a precise initial weight (W_5_). Then the sample was sealed in a retort pouch and boiled for 5 min. Following cooling, the fish muscle was reweighed after surface moisture removal (W_6_). Cooking loss was determined with Equation (3):

(3) $$\mathrm{Cooking}\;\mathrm{loss}\;(\%)=\frac{W_{5}-W_{6}}{W_{5}}\times 100\;$$

#### 2.3.6. Texture Profile Analysis

Texture profile analysis (TPA) was performed according to the method described previously with some modifications [22]. Uniform cubes of freshwater drum muscle samples were measured using a Texture Analysis XTPlus (Stable Micro Systems, Ltd., London, UK) equipped with a 5 mm diameter cylindrical probe (P/5). The instrument was calibrated according to the manufacturer’s specifications prior to analysis. The testing protocol was established with the following parameters: pre-test speed of 2.0 mm/s, test speed of 1.0 mm/s, post-test speed of 2.0 mm/s, trigger force of 5 g, compression distance of 60% strain, and inter-cycle interval of 10 s. Texture measurement was made on six cubes from each sample. Six fundamental texture parameters were derived from the force–time curves, including hardness, springiness, cohesiveness, gumminess, chewiness, and resilience.

### 2.4. Low-Field Nuclear Magnetic Resonance Analysis

Moisture distribution and proportions of the freshwater drum meat samples were evaluated using a Niumag benchtop pulsed NMR analyzer (Niumag Corporation, Shanghai, China). Following the method as previously reported with some modification [23], thawed freshwater drum meat samples were wrapped with polyethylene films and inserted into 60 mm NMR tubes for analysis. Transverse relaxation times (T_2_) were measured using the Carr–Purcell–Meiboom–Gill (CPMG) sequence with the following parameters: spectral width of 100 kHz, waiting time of 3000 ms, number of scans of 4, 180° pulse width of 14 μs, echo time of 0.1 ms, and number of echoes of 800. The acquired NMR relaxation data were processed using Nuimag Multi-Exp-Inv analysis software V3.0 for multi-exponential fitting. This revealed four distinct water populations characterized by T_21_ (bound water) with relative proportion P_21_ (%), T_22_ (immobilized water) with relative proportion P_22_ (%), and T_23_ (free water) with relative proportion P_23_ (%).

### 2.5. Muscle Histological Analysis

Muscle histology was evaluated following the reported method with minor modifications [24]. Thawed freshwater drum muscle samples were fixed in 4% paraformaldehyde for 24 h. The fixed tissues were then processed through a standard paraffin-embedding protocol: dehydration in graded ethanol series, clearing in xylene, and infiltration with paraffin. Embedded samples were sectioned at 5 μm thickness, mounted on glass slides, and stained with hematoxylin and eosin (H&E). Stained sections were dehydrated through an alcohol–xylene series and permanently mounted with neutral balsam. Microstructural evaluation was performed using an ECLIPSE Ci-L bright field microscope (Nikon, Tokyo, Japan), and digital images were captured with a DS-Fi3 camera system (Nikon).

### 2.6. Myofibrillar Protein Extraction

Myofibrillar protein extraction was performed according to the method as described previously [25]. Briefly, the samples were homogenized with 20 mL of ice-cold extraction buffer (pH 7.0, containing 0.1 M NaCl, 2 mM MgCl_2_, 1 mM EGTA, and 10 mM NaH_2_PO_4_) for 30 s, followed by centrifugation at 2000× *g* for 15 min at 4 °C. After discarding the supernatant, the pellet was washed three times under identical conditions. The precipitate was then resuspended in 0.1 M NaCl, homogenized for 30 s, and centrifuged again (2000× *g*, 15 min, 4 °C). Following three additional NaCl washes, the mixture was filtered through four layers of sterile gauze, adjusted to pH 6.0 with 0.1 M HCl, and centrifuged (2000× *g*, 15 min, 4 °C) to obtain purified myofibrillar proteins. The extracted proteins were maintained on ice (0–4 °C) and utilized within 24 h to ensure stability.

### 2.7. Fluorescence Spectral Analysis

For fluorescence characterization, the obtained myofibrillar protein solution was diluted to 0.5 mg/mL using 0.02 M phosphate buffer (pH 6.0, 0.6 M NaCl). Spectral analysis was performed with an Agilent Cary Eclipse fluorescence spectrophotometer (Agilent, CA, USA), recording emission spectra from 300 to 500 nm using an excitation wavelength of 290 nm. Prior to sample measurements, a phosphate buffer blank was analyzed for background correction to ensure accurate spectral data acquisition.

### 2.8. Statistical Analysis

All experiments were conducted with a minimum of three independent replicates, with results expressed as mean ± standard deviation (SD). Statistical evaluation was conducted using SPSS Statistics 26.0 (IBM SPSS Inc., Chicago, IL, USA). One-way analysis of variance (ANOVA) was applied to determine significant differences among treatment groups, followed by Duncan’s multiple range test for post hoc comparisons. Statistical significance was established at *p* < 0.05 for all analyses.

## 3. Results and Discussion

### 3.1. Changes in Freezing Curves

**Figure 1:**
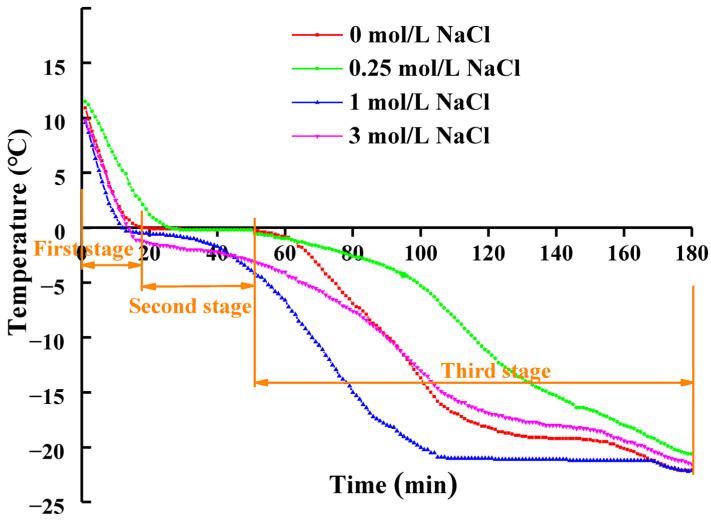
Freezing curves of freshwater drum (*Aplodinotus grunniens*) muscle meat centers under various curing concentrations (0, 0.25, 1, and 3 mol/L NaCl). The three characteristic freezing stages are delineated using the control group (0 mol/L NaCl) as a representative example.

The freezing curves of cured and uncured freshwater drum fish meat are presented in Figure 1, demonstrating three distinct freezing stages for all samples. The first stage is characterized by a rapid decrease in the core temperature of freshwater drum fish meat. The second stage, corresponding to the phase transition period, showed a markedly slower temperature change compared to the initial rapid cooling phase. As illustrated in Figure 1, the freezing point depression caused by dissolved NaCl lowered the temperature at which ice crystallization commenced. This stage represents the nucleation phase and is termed the “maximum ice crystal formation zone” (MIFZ). It was also found that the uncured samples required over 30 min to pass through the MIFZ, whereas the 0.25 mol/L NaCl-treated samples completed this phase in approximately 20 min. Notably, samples with higher salt concentrations (1 mol/L and 3 mol/L NaCl) exhibited a substantially shorter MIFZ duration, completing the phase in less than 10 min, which can be attributed to the enhanced freezing point depression resulting from the increased solute concentration. The third stage represents a sustained temperature decline phase, exhibiting a more gradual cooling rate compared to the initial rapid freezing stage.

Previous studies have established that extended ice crystal formation during freezing leads to the development of larger, non-uniformly distributed ice crystals that cause substantial mechanical damage to muscle cell ultrastructure, ultimately resulting in significant quality deterioration of meat products [26]. Our experimental findings demonstrate that pre-freezing NaCl treatment significantly shortens the ice crystal formation duration. This accelerated freezing process appears to be mediated by enhancing retention of unfrozen water within the protein matrix and effectively suppressing ice crystal growth. These phenomena may be structurally explained by NaCl-induced conformational changes in muscle proteins, particularly the promotion of β-sheet secondary structures and development of an organized three-dimensional macromolecular network [27].

### 3.2. Changes in Quality Characteristics

#### 3.2.1. Changes in Water Content and Salt Content

The effect of salting conditions on the water and salt content of freshwater drum meat is displayed in Table 1. The initial water content of the uncured fish meat was 71.26 ± 0.22%. As the NaCl concentration increased, a corresponding decrease in water content was observed. Notably, the water content was significantly reduced in the groups treated with 1 and 3 mol/L NaCl, reaching 70.47 ± 0.35% and 68.64 ± 0.50%, respectively. The decrease in water content is attributed to the mass transfer between water and salt during salting. When the fresh filets were immersed in the salt solutions, the high osmotic pressure drove the migration of tissue water out of the filets to equilibrate the internal and external osmotic pressures [28].

Salt concentration was identified as the main factor influencing the diffusion of salt and moisture. As shown in Table 1, the NaCl content in the muscle tissue increased with rising salt concentration in the treatment solution. The NaCl content reached 0.84 ± 0.06%, 2.92 ± 0.08%, and 8.46 ± 0.12% in the 0.25, 1, and 3 mol/L NaCl-pretreated groups, which were significantly higher than that of the uncured group (0.31 ± 0.01%). These findings were consistent with reports on salted tuna meat, in which higher salt concentrations led to increased NaCl uptake in the tissue due to increased osmotic pressure differences between the salt solutions and muscle tissue [4]. The decrease in water content and increase in salt uptake at higher NaCl concentrations not only altered the muscle’s osmotic balance but also affected subsequent parameters such as water activity, pH, and water-holding capacity, which are discussed below.

#### 3.2.2. Changes in Water Activity

The impact of curing treatment on water activity (aw) in frozen–thawed freshwater drum muscle is presented in Table 1. The results demonstrate a significant concentration-dependent reduction in a_w_ (*p* < 0.05), with values decreasing from 0.99 ± 0.00 to 0.94 ± 0.01 as the salt concentration increased from 0 to 3 mol/L. Our findings align with previous research on salt-mediated water activity reduction in meat products. Specifically, Demirhan Er and Demirhan demonstrated that marination with varying salt concentrations significantly influenced beef quality parameters, reporting notably lower water activity (aw) values in treated samples versus controls [29]. The aw reflects the binding strength between water and meat products as well as the availability of water for microbial utilization, while also influencing food safety and storage stability. The observed decrease in aw was attributed to the binding of Na^+^ and Cl^−^ ions with free water molecules in freshwater drum meat as NaCl concentration increased, resulting in reduced available free water following the freeze–thaw cycle. Additionally, microbial growth potential is fundamentally governed by aw. A reduction in aw selectively inhibits microbial proliferation, progressively limiting the spectrum of microorganisms capable of growth [30].

#### 3.2.3. Changes in pH Values

As displayed in Table 1, a significant salt concentration-dependent decrease in fish muscle pH (*p* < 0.05). While control samples maintained a pH of 7.09 ± 0.10, treatment with 0.25, 1, and 3 mol/L NaCl solutions reduced pH values to 6.73 ± 0.07, 6.80 ± 0.12, and 6.58 ± 0.07, respectively. This observation is consistent with established literature reporting a pH decrease in haddock (*Melanogrammus aeglefinus*) meat with increasing salt concentration. The underlying mechanism involves increased ionic strength and salt ion–protein interactions, which expose previously buried charged and/or hydrophilic groups, thereby altering the overall pKa of the protein matrix [17].

#### 3.2.4. Changes in Color Parameter

The color parameters of cured and uncured freshwater drum meat are also shown in Table 1. The results demonstrated significant variations in L* values across salt concentrations (*p* < 0.05), while a* and b* values remained statistically unchanged. L* values initially increased at lower salt concentrations (55.42 ± 2.60 at 0.25 mol/L NaCl and 52.77 ± 1.33 at 1 mol/L NaCl), then showed a significant decrease (*p* < 0.05) to 46.47 ± 1.45 at the highest concentration (3 mol/L NaCl). Our results corroborate Petracci et al.’s observations of salt-induced L* depression in meat. The mechanism involves surface dehydration from osmotic effects, which reduces interfacial water content and diminishes specular reflectance, collectively lowering L* values [31]. Additional studies demonstrated that high-salt curing reduces meat L* values through combined moisture loss and myosin denaturation [32].

#### 3.2.5. Changes in Water-Holding Capacity

**Figure 2:**
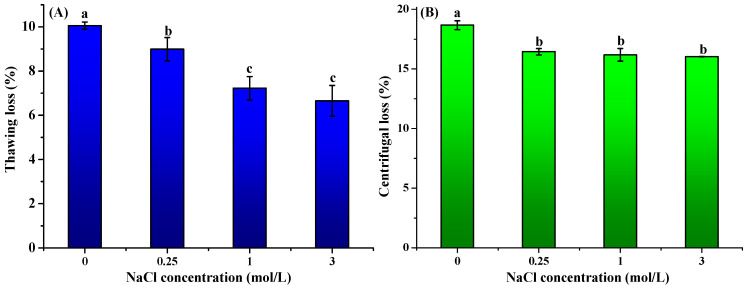

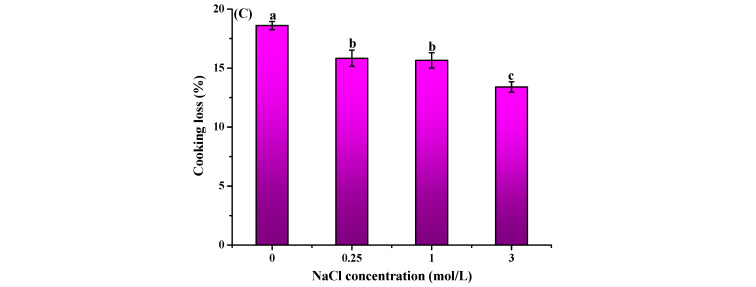
Effect of curing treatment (0, 0.25, 1, and 3 mol/L NaCl) on the thawing loss (**A**), centrifugal loss (**B**), and cooking loss (**C**) of frozen–thawed freshwater drum meat (*Aplodinotus grunniens*). Values with different lowercase letters denote statistically significant differences between groups (*p* < 0.05).

The impact of curing treatment on the water-holding capacity (WHC) of frozen–thawed freshwater drum (*Aplodinotus grunniens*) meat was presented in Figure 2. NaCl pretreatment significantly reduced (*p* < 0.05) thawing, centrifugal, and cooking losses compared to untreated controls. Compared to the untreated control (10.05 ± 0.17%), the thawing loss declined to 8.99 ± 0.53%, 7.22 ± 0.53%, and 6.65 ± 0.70% after pretreatment with 0.25, 1.0, and 3.0 μmol/L NaCl. Cooking loss showed maximal reduction of 16.03% at 3.0 mol/L NaCl, which was significantly lower than the untreated group (18.67 ± 0.37%). Centrifugal loss also exhibited a 5.21% decrease at the highest salt concentration (3.0 μmol/L NaCl) as compared to the control group. The present study demonstrates that NaCl pretreatment prior to freezing significantly enhances the WHC of freshwater drum meat. These findings align with the previous research that revealed similar WHC improvements in light-salt-treated grass carp (*Ctenopharyngodon idellus*). The changes in WHC might be attributed to salt-induced solubilization of myofibrillar proteins that increase available hydration sites and electrostatic repulsion between chloride-ion-loaded proteins that create larger interstitial spaces for water retention [33].

#### 3.2.6. Changes in Texture Profile

**Figure 3:**
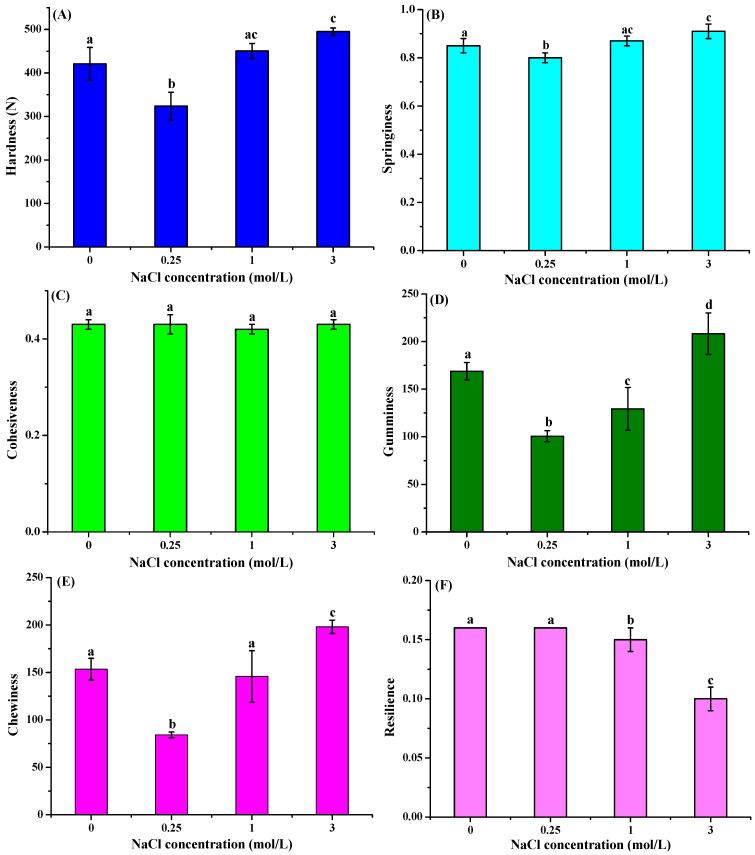
Effect of curing treatment (0, 0.25, 1, and 3 mol/L NaCl) on the hardness (**A**), springiness (**B**), cohesiveness (**C**), gumminess (**D**), chewiness (**E**), and resilience (**F**) of the frozen–thawed muscle of freshwater drum meat (*Aplodinotus grunniens*). Values with different lowercase letters denote statistically significant differences between groups (*p* < 0.05).

Figure 3 presents the significant effects of salt curing on the texture properties of frozen freshwater drum muscle. Hardness, gumminess, and chewiness exhibited a concentration-dependent increase with rising NaCl concentrations. Hardness initially decreased significantly from 421.04 N (control) to 323.81 ± 32.00 N at 0.25 mol/L NaCl (*p* < 0.05), then showed a concentration-dependent increase to 450.45 ± 17.18 N (1.0 mol/L) and 494.94 ± 8.48 N (3.0 mol/L). Gumminess displayed an initial reduction to 100.47 ± 5.73 at 0.25 μmol/L NaCl as compared to the control group, followed by progressive recovery to 129.19 ± 22.54 at 1 mol/L and ultimately exceeding control values at 208.24 ± 21.79 (3 mol/L NaCl). Chewing also followed an analogous pattern. No significant differences were observed in cohesiveness among different NaCl concentrations. Resilience showed significant decreases specifically in the 1 and 3 mol/L NaCl treatment groups. In contrast to other texture parameters, springiness showed no significant variation among different NaCl concentrations. These findings are consistent with previous studies demonstrating dose-dependent effects of brine salting on grass carp hardness [28]. The observed hardness increase may be attributed to the combined effects of ions from salt and the low pH condition in muscle tissue, which corresponded to the results obtained from Table 1. It has also been revealed that salt-induced myofibrillar protein denaturation leads to reduced gel-forming capacity and increased tissue toughness [34]. Our findings demonstrate that NaCl pretreatment significantly enhances the textural quality of freshwater drum meat. The improvement in hardness and chewiness observed at higher NaCl concentrations is consistent with the increased water-holding capacity.

### 3.3. Changes in Moisture Distribution and Proportions

**Figure 4:**
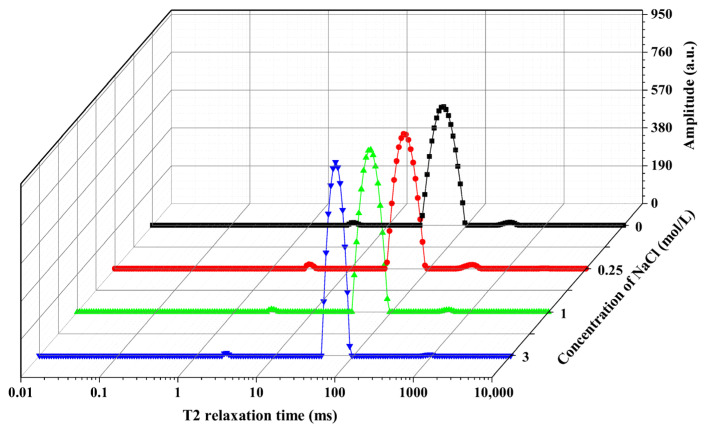
Effects of different NaCl concentrations (0, 0.25, 1, and 3 mol/L) on the T_2_ relaxation spectra of frozen–thawed freshwater drum fish meat.

Low-field nuclear magnetic resonance (LF-NMR) technology has been widely employed to analyze the state of the water in fish muscle, primarily by employing transverse relaxation time (T_2_) to characterize the constant proton (^1^H) relaxation properties [35]. Figure 4 shows the T_2_ water distribution profiles of frozen freshwater drum fish meat after thawing, as treated with different NaCl brine concentrations. Through multi-exponential fitting of the T_2_ distribution, three distinct peaks were identified that are considered to correspond directly to three distinct water components. The three relaxation components were assigned as follows: The first peak, designated as T_21_, appears between 1 and 10 ms and corresponds to bound water. The second peak, labeled T_22_, occurs between 30 and 100 ms and represents immobilized water. The third peak, observed between 200 and 1000 ms and denoted as T_23_, is attributed to free water [36].

Table 2 presents the changes in transverse relaxation time (T_2_) and the proportion of water in different states (P_2_) in freshwater drum marinated with different NaCl concentrations after freeze–thaw treatment. No significant difference was observed in T_21_ values between NaCl-treated freshwater and the non-marinated control group after freeze–thaw treatment. This may be due to the strong binding between water molecules and macromolecules. The T_22_ value of the samples marinated with 3 mol/L NaCl increased significantly compared to the non-marinated control group. The increased immobilized water mobility might be related to the high NaCl concentration-induced swelling of myofibrillar proteins, thereby enabling the retention of more water [33]. The variation trends of P_21_ (bound water) and P_22_ (immobilized water) in frozen–thawed freshwater drum muscle with respect to NaCl concentration were generally consistent with those observed for T_2_. Compared to the non-marinated control group (357.08 ± 0.00 ms), the T_23_ values of samples treated with NaCl at varying concentrations showed significant increases, reaching 519.05 ± 55.87 ms (0.25 mol/L NaCl), 517.37 ± 20.97 ms (1.0 mol/L NaCl), and 791.22 ± 132.12 ms (3.0 mol/L NaCl) (*p* < 0.05), respectively. Simultaneously, the proportion of free water (P_23_) decreased from 1.22 ± 0.21% to 0.58 ± 0.05%, 0.59 ± 0.04%, and 0.38 ± 0.11% (*p* < 0.05), corresponding to each treatment group. This phenomenon may be attributed to the migration of free water from the freshwater drum muscle into the brining solution or its conversion into immobilized water [33].

### 3.4. Changes in Muscle Histology

**Figure 5:**
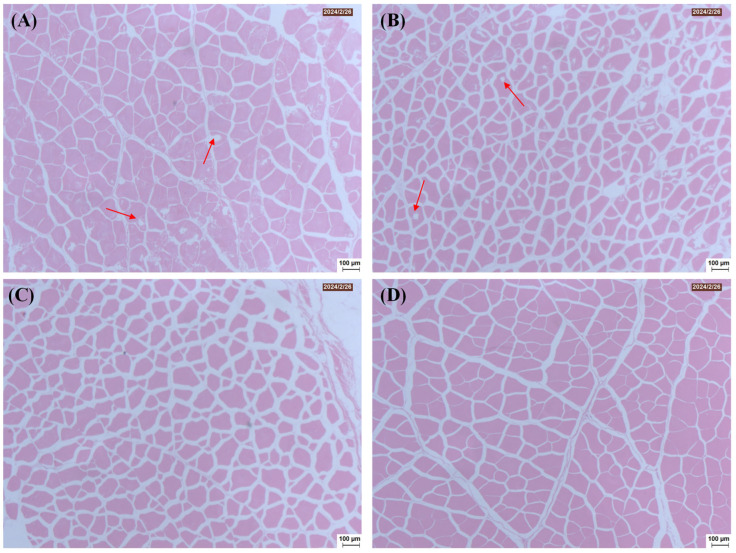
Effect of curing treatment (0 (**A**), 0.25 (**B**), 1 (**C**), and 3 (**D**) mol/L NaCl) on the histological microstructure of frozen–thawed freshwater drum meat.

The structural changes in the muscle tissue of salt-treated freshwater drums following freezing and thawing were further investigated through light microscopy. As shown in Figure 5, the myofibrils of the fish meat treated with 1 mol/L NaCl and 3 mol/L NaCl appeared in a typical irregular polygonal shape, neat arrangement, and a compact, smooth-surfaced structure as compared to the uncured group, suggesting that freezing was associated with less structural damage to the microstructure of the cured samples. The microstructure of muscle is governed by the interactions between proteins and water molecules. This phenomenon might be due to the dissolution of myofibrillar proteins and the swelling of myofilament lattices induced by salting, which is closely related to the improved gumminess, chewiness and water-holding capacity [13]. It should be pointed out that pores formed between muscle fibers in both uncured samples and those cured with 0.25 mol/L NaCl. As shown in Figure 1, these samples formed more ice crystals during the freezing process. The formation of pores is primarily attributed to the mechanical damage caused by ice crystals to the muscle, as well as the alterations in protein properties, leading to the deterioration of the tissue structure and consequently reducing the quality and textural properties of the samples [37]. This is consistent with the results shown in Figure 3 and Figure 4. The above findings indicate that salt curing under appropriate conditions can enhance the recovery ability of the tissue structure in thawed freshwater drum meat, thereby helping the samples maintain higher water-holding capacity and textural properties.

### 3.5. Changes in Fluorescence Spectrum

**Figure 6:**
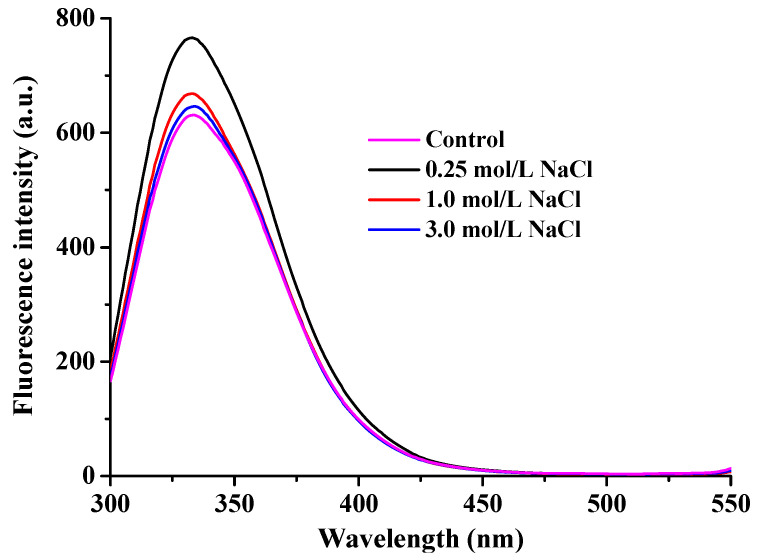
Effect of NaCl concentration (0, 0.25, 1, and 3 mol/L) on the fluorescence intensity of myofibrillar proteins in frozen–thawed freshwater drum meat.

The intrinsic fluorescence of proteins is derived from three aromatic amino acid residues—tryptophan, tyrosine, and phenylalanine—which are sensitive to the polarity of their microenvironments. As the most commonly used intrinsic fluorescent probe, tryptophan residues are located in both the head and rod domains of myofibrillar proteins. Measurements of their fluorescence intensity provide a sensitive means to characterize conformational changes in protein structure [38]. As depicted in Figure 6, the maximum fluorescence emission of myofibrillar proteins in freshwater drum was observed at approximately 330–340 nm. Salt treatment resulted in a slight blue shift from 335 nm (control and 0.25 mol/L NaCl-treated group) to 334 nm (1 mol/L NaCl) and 333 nm (3 mol/L NaCl). These results indicate that a portion of the tryptophan residues was exposed to a hydrophilic microenvironment [39]. Additionally, with increasing NaCl concentration, the fluorescence intensity of myofibrillar proteins in freshwater drums initially increased and then decreased after freeze–thaw cycling. This finding is consistent with previously reported frozen–thawed Enshi pork meat following curing [40]. It has been reported that protein unfolding leads to the gradual exposure of tryptophan residues to the polar aqueous solvent, resulting in fluorescence quenching. Collectively, these fluorescence changes indicate that NaCl helps preserve the tertiary structure of myofibrillar proteins in freshwater drums against freeze-induced damage [41]. The fluorescence spectra corroborate the histological findings, suggesting that NaCl pretreatment mitigated protein denaturation and maintained the tertiary structure of myofibrillar proteins, thereby contributing to the enhanced texture and reduced moisture loss observed in previous analyses

## 4. Conclusions

This study demonstrates that NaCl pickling pretreatment, particularly at 3 mol/L, effectively improves the quality of frozen–thawed freshwater drum (*Aplodinotus grunniens*) meat through the following synergistic pathways. First, it can accelerate freezing kinetics, shortening transit time through the maximum ice crystal formation zone and reducing structural damage. Second, pickling pretreatment optimizes water redistribution, markedly improving water-holding capacity while lowering water activity. Third, high salt concentrations preserve microstructural integrity and textural properties, accompanied by reduced pH. Our results demonstrate that while pretreatment with NaCl at concentrations ≥ 1 mol/L effectively preserves the post-thaw quality of freshwater drums, 1 mol/L NaCl concentration is more suitable for application due to the excessively high salt content resulting from the 3 mol/L NaCl treatment. It is important to note that global efforts are underway to reduce sodium intake due to its association with increased risk of chronic diseases. Therefore, future research should prioritize strategies for lowering NaCl content in freshwater drum products. Furthermore, systematic investigation into the influence of NaCl levels on sensory qualities (e.g., taste and smell) is essential for product optimization and consumers.

## Figures and Tables

**Table 1:** Effects of varying NaCl concentrations (0, 0.25, 1, and 3 mol/L) on water content, salt content, water activity, pH, and color of frozen–thawed freshwater drum (*Aplodinotus grunniens*) muscle.

Quality Indicators	Uncured	0.25 mol/L NaCl	1.0 mol/L NaCl	3.0 mol/L NaCl
**Water content (%)**	71.26 ± 0.22 ^a^	71.08 ± 0.34 ^ab^	70.47 ± 0.35 ^b^	68.64 ± 0.50 ^c^
**Salt content (%)**	0.31 ± 0.01 ^a^	0.84 ± 0.06 ^b^	2.92 ± 0.08 ^c^	8.46 ± 0.12 ^d^
**aw**	0.99 ± 0.00 ^a^	0.98 ± 0.00 ^a^	0.96 ± 0.00 ^b^	0.94 ± 0.01 ^c^
**pH**	7.09 ± 0.10 ^a^	6.73 ± 0.07 ^b^	6.80 ± 0.12 ^b^	6.58 ± 0.07 ^c^
L*	50.04 ± 1.40 ^a^	55.42 ± 2.60 ^b^	52.77 ± 1.33 ^ab^	46.47 ± 1.45 ^c^
a*	−2.53 ± 0.12 ^a^	−2.40 ± 0.10 ^a^	−2.25 ± 0.38 ^a^	−2.65 ± 0.17 ^a^
b*	−2.64 ± 0.39 ^a^	−2.17 ± 0.30 ^a^	−1.79 ± 0.72 ^a^	−2.47 ± 0.21 ^a^

Note. Different lowercase letters in a row indicate significant differences (*p* < 0.05) between the two groups.

**Table 2:** Changes in water transverse relaxation time (T_2_) and proportions (P_2_) of different water states in frozen–thawed freshwater drum meat cured with different NaCl concentrations (0, 0.25, 1, and 3 mol/L).

Quality Indicators		Uncured	0.25 mol/L NaCl	1.0 mol/L NaCl	3.0 mol/L NaCl
**T_2_/ms**	**T_21_**	3.34 ± 0.35 ^a^	4.81 ± 1.14 ^a^	4.56 ± 1.39 ^a^	4.82 ± 2.77 ^a^
	**T_22_**	48.83 ± 1.98 ^a^	46.62 ± 1.85 ^a^	50.05 ± 4.10 ^a^	60.14 ± 2.44 ^b^
	**T_23_**	357.08 ± 0.00 ^a^	519.05 ± 55.87 ^b^	517.37 ± 20.97 ^b^	791.22 ± 132.12 ^c^
	**P_21_**	1.29 ± 0.80 ^a^	1.66 ± 0.43 ^a^	1.26 ± 0.24 ^a^	1.12 ± 0.67 ^a^
**P_2_/%**	**P_22_**	97.47 ± 0.79 ^a^	97.76 ± 0.42 ^a^	98.13 ± 0.72 ^a^	98.50 ± 0.76 ^a^
	**P_23_**	1.22 ± 0.21 ^a^	0.58 ± 0.05 ^b^	0.59 ± 0.04 ^b^	0.38 ± 0.11 ^b^

Note. Different lowercase letters in a row indicate significant differences (*p* < 0.05) between the two groups.

## Data Availability

The original contributions presented in this study are included in the article. Further inquiries can be directed to the corresponding authors.
